# Lithium in the episode and suicide prophylaxis and in augmenting strategies in patients with unipolar depression

**DOI:** 10.1186/s40345-017-0080-x

**Published:** 2017-05-08

**Authors:** Mohammed T. Abou-Saleh, Bruno Müller-Oerlinghausen, Alec J. Coppen

**Affiliations:** 1 St George’s, University of London, Cranmer Terrace, London, SW17 0RE UK; 2 Drug Commission of the German Medical Association, Freie Universität Berlin, Charité Universitäts-Medizin, Berlin, Germany; 3 5 Walnut Close, Epsom, Surrey, KT18 5JL UK

**Keywords:** Lithium, Long-term-prophylaxis, Unipolar depression, ECT, Antisuicidal effect, Augmentation

## Abstract

**Background:**

Depressive disorders are a leading cause of the global burden of disease and are associated with high recurrent often continuing morbidity and high excess mortality by suicide and cardiovascular disease. Whilst there are established, effective and cost-effective treatments for depression, their long-term management is often neglected: there is continuing controversy over the case of need for long-term treatment including lifelong treatment and safety issues.

**Objective and methods:**

In this narrative review, we critically examine the evidence for the effectiveness and safety of lithium salts in the long-term management of unipolar depression. We refer to existing recent international guidelines as well as the scientific literature selectively and against the background of our longstanding experience with patients suffering from unipolar depression who are often under treated or inappropriately treated.

**Results and discussion:**

According to many studies mostly dating back to the 1970/1980s, lithium is efficacious in the prophylaxis of unipolar depression particularly depression with melancholia and delusional depression and showing a clearly episodic course. Also the efficacy of lithium maintenance treatment following recovery by ECT has been clearly shown. Moreover, convincing evidence exists that lithium has added value and benefit for its unique anti-suicidal effects as well as reducing mortality by other causes. The anti-suicidal effect has been convincingly demonstrated in bipolar as well as in unipolar patients. Nevertheless its use in the management of patients with unipolar depression has not been properly recognized by a majority of textbooks and guidelines. Whilst it has been well considered as an effective treatment for depression that has not responded to antidepressants as an adjunct treatment, also called augmentation, it has been much less recommended for the prevention of recurrent episodes of unipolar depression. One of the reasons for this neglect is the blurring of the diagnosis “unipolar depression” by modern diagnostic tools. Lithium will hardly work in a patient with “unipolar depression spectrum disease”.

**Conclusions:**

We conclude that lithium is an effective prophylactic treatment for carefully selected patients with unipolar depression and is safe when prescribed in recommended doses/plasma lithium levels and with regular, careful monitoring. We propose that lithium prophylaxis can be indicated in patients with unipolar depression and that the occurrence of 2 episodes of depression within 5 years is a practical criterion for starting lithium prophylaxis particularly in severe depression with psychotic features and high suicidal risk. Furthermore, an indication might be considered especially in unipolar patients in whom a bipolar background is suspected. In some cases, lithium prophylaxis may be recommended after a single episode of depression that is severe with high suicidal risk and continued life-long.

## Background and methods

The serendipitous discovery of lithium and its introduction to psychiatry in 1949 has provided one of the most dramatic developments in psychiatric practice. Indeed it has antedated the introduction of chlorpromazine in 1957 and imipramine in 1962. Moreover, its established efficacy in the management of manic-depressive psychosis affirmed Kraeplin’s distinction between schizophrenia and manic-depressive psychosis and later on the distinction between bipolar and unipolar affective disorder. Its efficacy in the management of affective disorders has been established by numerous high-quality controlled studies which showed that the use of lithium substantially reduces the morbidity and mortality of recurrent affective disorders (Coppen 1994) including unipolar depression (Coppen and Abou-Saleh 1983; Coppen 2000a, b; Guzzetta et al. 2007; Bschor 2014; Lewitzka et al. 2015a).

This narrative review focuses on the use of lithium in the management of patients with unipolar depression. It selectively reviews the evidence for the effectiveness of lithium as an augmentation treatment to antidepressants, continuation medication after ECT and importantly as prophylactic treatment in unipolar depression with reference to its unique antisuicidal effects. As to the selected scientific literature we are referring to, we took advantage of the recently published evidence-based guidelines by the World Federation of Societies of Biological Psychiatry (WFSBP) on the maintenance treatment of major depressive disorder (Bauer et al. 2015).

## The burden of unipolar depression

Depressive disorders are a leading cause of the global burden of disease and are associated with high recurrent often continuing morbidity including physical multi-morbidity and high mortality by suicide and other causes. Worldwide, it is estimated that 350 million people are affected. Data from the Global Burden of Diseases, Injuries, and Risk Factors Study 2010 reported that depressive disorders accounted for 40.5% of DALYs caused by mental and substance use disorders (Whiteford et al. 2013). The World Health Organization projections estimate that it will be the leading cause of disease burden worldwide by 2030. The estimated median treatment gap for depression is 56.3% ranging from 45.4% in Europe to 70.2% the Eastern Mediterranean Region (Kohn et al. 2004). Worldwide, depressive disorders contribute to 2.2 million excess deaths in 2010 by suicide and comorbid disease such as cardiovascular disease (Patel et al. 2016).The WHO Mental Health Action Plan 2013–2020 aims for a 10% reduction in the suicide rate and health-care services need to incorporate suicide prevention as a core component (WHO 2013). The plan advocates the implementation of evidence-based interventions in at risk populations including people with mood disorders but there is no mention of the effectiveness of lithium for the prevention of suicide. Moreover, the WHO report on suicide prevention has not mentioned the role of lithium among pharmacological treatments (WHO 2014). However, WHO’s Mental Health Gap Action Programme, which was launched in 2008, includes suicide as one of the priority conditions and provides evidence-based technical guidance to expand service provision in countries.

## The course of unipolar depression

Unipolar depression in its classic form is an episodic disorder (Angst et al. 1973, 1986) with a high persisting morbidity. The first episode starts commonly around 42 years of age and can last for a period of up to 12 months or more. The episode will then remit and the patient can be symptom free for a period of several years before it is followed by a further episode in a substantial proportion of patients. Many patients will have multiple episodes; the length of an episode usually does not vary but the interval between the episodes decreases. After 3 or 4 episodes, patients can spend a considerable proportion of their lives (on average 30%) with a depressive episode (Coppen et al. 1971). (It should, however, be remembered that the origin of these observations date back to the 1960 ies/1970 ies when the Kraepelian type of depression was much more frequently presented in psychiatric hospitals). The population attributable risk for suicide in mood disorders is 26% for males and 32% for females (Li et al. 2011). In clinical populations and by 18 months, 10% of individuals with unipolar depression attempt suicide when ill particularly among patients with previous attempts, female patients, those with poor social support, and those aged 40 years or less (Holma et al. 2014), Similar rates were observed over 5 years in primary care (Riihimäki et al. 2015). The incidence of suicide attempts during major depressive episodes was 21-fold and fourfold during partial remission compared with full remission (Holma et al. 2014).

Unipolar depression is not a uniform, homogenous entity. Particularly in recent decades, the introduction and general use of modern diagnostic systems such as ICD-10 or DSM -IV/5 have broadened its definition and blurred the picture and course of formerly defined unipolar depression favouring terms such as “spectrum disease”, “mixed states” including a variety of psychiatric comorbidities (Ghaemi 2008).

Furthermore, it has been shown by several researchers that there exists a background of “bipolarity” in quite a number of patients with severe “major depressive disorder” or “unipolar depression” and also in “atypical depression” and “pseudounipolars” (Marneros and Angst 2002). Particularly, those patients with early onset and a high number of episodes in early life are at risk to convert to bipolar disorder. (Akiskal et al. 1989, 2000; Angst et al. 2013; Lojko et al. 2015; Rihmer et al. 2016; Park and Lee 2016).

A recent review of the evidence for the mood spectrum model in light of DSM-5 showed that the mood spectrum model approach has demonstrated its usefulness mainly in 3 areas: (1) Patients with the so-called “pure” unipolar depression that might manifest hypomanic atypical and/or sub-threshold aspects; (2) Spectrum features that are clinically relevant, because they might manifest in waves during the lifespan, sometimes together, sometimes alone, sometimes reaching the severity for a full-blown disorder, sometimes interfering with other mental disorders or complicating the course of somatic diseases; and (3) Features of “psychomotor disturbances”, “mixed instability” and “suicidality” delineate subtypes of patients characterized by the more severe forms of mood disorders, the higher risk for psychotic symptoms, and the lower quality of life after the remission of the full-blown-episode (Benvenuti et al. 2015). This heterogeneity should have considerable impact on the potential response to antidepressants or mood stabilizers such as lithium.

The Yale family study of delusional depression (DD) (Leckman et al. 1984; Weissman et al. 1984) supports a relationship of DD with bipolar disorder, but not as establishing DD as simply a subtype of bipolar disorder. The possibility of a unique, late-onset unipolar subgroup is suggested by the apparent bimodal distribution of the age of onset of DD, the occurrence of a dementia-like presentation only in the older age group and the association of recurrence and a treatment refractory course with older age. Another study (Coryell et al. 1986) also noted in a group of psychotic depressives that a point of rarity in the age distribution occurred at 50 years. The early-onset unipolar subgroup was characterized by an index episode of DD in the patient’s 20 and 30 s and a recurrent course.

A retrospective study of phenomenology and treatment course of delusional depression reported a high relapse rate of unipolar DD (83.3%) (Aronson et al. 1988). It was proposed that unipolar DD will require long-term maintenance pharmacologic treatment, as 40.7% of the relapses occurred during or shortly after a medication taper.

## Lithium augmentation in acute treatment of depression

Studies including randomized double-blind trials of lithium monotherapy for the acute treatment of depression have not established its efficacy compared with antidepressants (Bschor 2014). However, even with antidepressants, the overall response rate is modest. Lithium is able to optimize the response in hitherto poor responders. This special use of lithium is called augmentation.

Since the first report on the efficacy of lithium augmentation treatment for depression that has not responded to tricyclic antidepressants (Dé Montigny et al. 1981), numerous RCTs and meta-analyses demonstrated the efficacy of lithium augmentation. Meta-analysis by Crossley and Bauer (2007) included 10 placebo-controlled trials showing that lithium was not only more efficacious than placebo but had a relatively large effect size and number-needed-to-treat of 5. Only 2 of these RCTs were of SSRIs (Katona et al. 1995 and Baumann et al. 1996). The study by Katona and colleagues (1995) was the largest RCT (*N* = 62) examining response to adjunctive lithium inpatients receiving fluoxetine or lofepramine. Lithium or placebo was added on a double-blind basis for 6 weeks to the drug regime of 62 patients with major depressive illness (in both hospital and primary care settings) who had failed to respond to a controlled trial of fluoxetine or lofepramine. Response was seen more frequently in patients taking lithium than in those remaining on antidepressant alone. Rapid response to lithium augmentation was not consistently observed, but was evident in those maintained on adequate plasma lithium levels >or = 0.4 mmol/l. A recent systematic review and meta-analysis of lithium augmentation of tricyclic and second generation antidepressants in major depression was published by Nelson et al. (2014). Nine trials that included 237 patients were selected. Adjunctive lithium was effective with TCAs (7 contrasts) and with second generation agents (3 contrasts). Discontinuation due to adverse events was infrequent and did not differ between lithium and placebo. The meta-analysis was limited by the small size and number of trials and limited data for treatment-resistant patients. A comparison of lithium and T(3) augmentation following two failed medication treatments for depression in the STAR*D study showed that remission rates with lithium and T(3) augmentation for participants who experienced unsatisfactory results with two prior medication treatments were modest and did not differ significantly (Nierenberg et al. 2006).

It is concerning that despite the good evidence that lithium augmentation is clearly effective; it is not widely used for treatment of depression. A study of adjunctive treatments in a Veterans Administration medical setting found that of 53,807 depressed patients that received adjunctive treatment because of unsatisfactory response, 49.7% received a second antidepressant and 33.1% received an atypical antipsychotic (Valenstein et al. 2006). Only 1106 patients (2%) received lithium augmentation (Nelson et al. 2014). A survey of UK psychiatrists showed that only 12% of them have used lithium augmentation as first choice after the use of an antidepressant (Shergill and Katona 1997). It appears as if many patients with refractory depression are not receiving further treatment according to rational algorithms (Bschor 2014).

A recent survey of lithium prescribing patterns for a clinical audit of the quality of lithium monitoring, conducted by the Prescribing Observatory for Mental Health in the UK reported on 2776 patients with a diagnosis of affective disorder 31% of whom had non-bipolar affective disorder (Paton et al. 2010). Co-prescribing was common; 77% of those with non-bipolar affective disorders received an antidepressant, prescribing that is consistent with evidence-based treatment guidelines that may reflect difficulty managing the symptoms of affective disorders with lithium monotherapy. One in 10 patients of patients prescribed lithium had a sub-therapeutic blood level (<0.4 mmol/l) and so might have been at high risk of relapse.

A recent systematic review and network meta-analysis of the comparative efficacy, acceptability, and tolerability of augmentation agents in treatment-resistant depression showed that quetiapine, aripiprazole, thyroid hormone and lithium were significantly more effective but less tolerable than placebo (Zhou et al. 2015). A comprehensive meta-analysis of efficacy and safety outcomes in short-term trials of lamotrigine compared to placebo and other agents with antidepressant activity in patients with unipolar and bipolar depression showed that lamotrigine did not differ regarding efficacy on depressive symptoms, response, or remission from lithium (Solmi et al. 2016).

## Lithium as a continuation treatment following ECT

Electroconvulsive therapy (ECT) is an effective treatment for patients with severe, psychotic and medication-resistant depression. Although the immediate response to ECT is good, ample evidence exists that there is a high incidence of relapse in the months following treatment unless antidepressants are given as continuation therapy (Seager and Bird 1962).

In the first prospective study of lithium continuation therapy following ECT, a group of 38 patients who had responded to ECT were studied (Coppen et al. 1981; Abou-Saleh and Coppen 1988). The patients were randomly allocated to receive either placebo or lithium therapy for 1 year. The patients who received lithium spent significantly less time with a relapse (average 1.7 weeks over the year) than the placebo group (over 7.8 weeks). The difference was particularly marked during the second 6 months of the study when the lithium patients spent 0.2 weeks with a relapse compared to 5.6 weeks in the placebo group. Lithium thus appears to be a satisfactory continuation therapy after recovery from the acute episode. It was noted that the efficacy of lithium continuation treatment was more evident in the second 6 months of the trial indicating the need for continuation treatment beyond the commonly recommended 6 months. Further analysis of the data suggested that lithium may be as effective in preventing a recurrence (new episode) as continuation treatment to prevent early relapse of the ongoing episode of depression (Abou-Saleh 1987). The conclusion was that ECT is a very effective form of treatment of severe depression but must be accompanied by continuation therapy for its optimum effect; this continuation therapy should be maintained for up to 1 year after ECT. If the patient has had several attacks of depression, then long-term prophylaxis must be seriously considered.

Another placebo-controlled trial studied the efficacy of continuation pharmacotherapy with nortriptyline compared with a combination of nortriptyline and lithium in preventing post-ECT relapse over 24 weeks (Sackeim et al. 2001). Nortriptyline-lithium combination therapy had a marked advantage in time to relapse, superior to both placebo and nortriptyline alone. Over the 24-week trial, the relapse rate for placebo was 84%; for nortriptyline, 60% and for nortriptyline-lithium, 39%. All but one instance of relapse with nortriptyline-lithium occurred within 5 weeks of ECT termination, while relapses continued throughout treatment with placebo or nortriptyline alone. Medication-resistant patients, female patients, and those with more severe depressive symptoms following ECT had more rapid relapses. The conclusion was that without active continuation treatment, virtually all remitted patients relapse within 6 months of stopping ECT. Monotherapy with nortriptyline has limited efficacy. The combination of nortriptyline and lithium is more effective, but the relapse rate is still high, particularly during the first month of continuation therapy.

A recent consideration of the evidence for the efficacy of post-ECT lithium therapy in the prevention of relapse concluded that, “in the main, there is strong evidence that lithium can help prevent relapses in the first 6 months after index ECT. However, there are several unanswered questions about its use post-ECT, including optimal target blood level, duration of use, and concomitant antidepressant choice” (Rasmussen 2015). A very recently published RCT focussed on the comparison of a “medication only”(venlafaxine plus lithium) vs. an “continuation ECT plus medication” group observed over 24 weeks after an acute course of ECT. The results of the combined group were more favourable than those of the medication only strategy (Kellner et al. 2016).

## Lithium prophylaxis in unipolar depression

The first systematic study of lithium prophylaxis in recurrent affective illness using a “mirror” or “before and after” design was reported by Baastrup and Schou (1967). Before lithium treatment, the patients spent 3.88 months per year with an affective episode and this fell to 0.27 months per year during the period of lithium treatment. Other investigations using a similar ‘before and after design’ also produced similarly encouraging reports (Angst et al. 1973; Hullin et al. 1972). There followed prospective double-blind placebo-controlled studies in which patients were randomly allocated to receive either lithium or placebo for a fixed period during which time they were regularly assessed. Relapses were treated by conventional antidepressant measures, but the prophylactic measures were not changed. This enables a sensitive measure of benefit to be obtained either in terms of the time spent with an episode or as the “Affective Morbidity Index” (AMI). The AMI is a composite measure both of duration and severity of affective episodes (Coppen et al. 1973; Baethge et al. 2003), and is calculated by measuring the area under the curve made by joining all the points representing ratings of severity of affective disturbance at each assessment. Such a measure is invaluable in calculating reduction of morbidity where, as is often the case, there is not a complete abolition of morbidity.

Coppen et al. (1971) reported a highly significant reduction of morbidity in unipolar depressives in a prospective double-blind placebo-controlled trial in which patients received lithium or placebo over 27 months. The percentage of time spent with a depressive episode was 4.7% in the lithium group and 30% in the placebo group. The AMI was 0.12 in the lithium patients compared to 0.60 in those who received placebo. All these differences were statistically significant. No patient in the lithium group required ECT but almost half the patients in the placebo group required one or more courses of ECT.

The results of the trial were updated so that they could be examined in intent-to-treat analysis of the original cohort using the global assessment based on a five-point scale (Coppen 2000a, b). The subgroup of patients with unipolar depression who were treated with lithium did significantly better at the end of the study than the placebo-treated patients. The results in unipolar patients were comparable with those obtained in studies of maintenance antidepressant treatment of depressed patients (Kupfer et al. 1992) which are generally conducted in patients who have responded to acute antidepressant medication.

The first meta-analysis on the acute and long-term effects lithium in unipolar depression found an “impressive effect” of lithium vs. placebo in RCTs over 5 months to 3 years and also in uncontrolled studies (Souza and Goodwin 1991). The second systematic review of the efficacy of lithium treatment compared to placebo in the prevention of relapse in recurrent mood disorders indicated that in unipolar depression, the evidence of efficacy is less robust than in bipolar disorder (Burgess et al. 2001). Descriptive analysis, however, showed that assessments of general health and social functioning generally favoured lithium. However, it should again be emphasized that the bulk of patients selected for those studies were recruited in the 1970 ies and early 1980 ies. Thus, their psychopathology most likely was different from “modern” patients with various types of “depression spectrum disease”.

The third systematic review of the efficacy of lithium treatment compared to antidepressants in the long-term treatment of unipolar depression was published by Cipriani et al. (2006). Eight randomized, controlled trials involving 475 patients were included. There was a statistically significant difference in favour of lithium (relative risk (RR) fixed effect 0.34, 95% Cl 0.14–0.82) which was significantly superior to antidepressants in preventing relapses that required admission to hospital. The authors concluded that “there was adequate efficacy evidence for lithium or antidepressants preventing relapse in unipolar affective disorder depression, however their relative efficacy was unknown. When considering lithium or antidepressant long-term therapy, patients and clinicians should take into account the patient’s clinical history, the side-effects and the individual’s likely adherence to the recommended treatment regime”.

Long-term naturalistic studies strongly supported the effectiveness of lithium prophylaxis for unipolar depression disorder in clinical practice.

An early study explored the response to lithium therapy in 95 patients with unipolar depression. Response to lithium showed a linear relationship to Newcastle Scale scores (measure of the degree of endogeneity) in these patients. The AMI of patients with endogenous and psychotic depression was very low indicating an excellent response to prophylactic lithium which was similar to AMI of patients with bipolar disorder (Abou-Saleh and Coppen 1983). Similar findings were reported after the adoption of lower doses/levels of lithium in 1983 (Coppen and Abou-Saleh 1988).

Response to prophylactic lithium was studied in relation to clinical and psychological characteristics in a large series of patients with recurrent affective disorders (Abou-Saleh and Coppen 1986). Unipolar patients with more endogenous illnesses and those with pure familial depressive disease had more favourable responses than those with less endogenous illnesses and those with sporadic and depression spectrum disorders. Good responders showed generally less personality disturbance on a variety of measures than fair-to-poor responders. Response to lithium over the first 6 months of treatment in unipolar illness was strongly associated with long-term response.

Other long-term naturalistic studies of the effectiveness of long-term lithium prophylaxis in unipolar depression during an average follow-up period of 6.7 years reported a significant decline in the number of days spent in hospital and a low AMI was observed in these carefully monitored and documented patients from a specialized lithium clinic, who originally most likely had been diagnosed according to Kraepelian tradition (Baethge et al. 2003).

The meta-analysis by Kim et al. (1990) though on the basis of a rather restricted number of patients showed that combining lithium and imipramine leads to better episode prevention than either lithium or imipramine alone. Also the wfsbp-guideline (Bauer et al. 2015) recommends a combination of lithium and antidepressant in patients who fail to remain well on either drug alone

## Indications for commencing prophylaxis in unipolar depression

A crucial decision for the clinician is when to start lithium prophylaxis. A detailed and profound discussion of this topic was published by Angst (1980). He analysed the course of the illness in 159 unipolar patients probably diagnosed in a traditional European manner and decided that it would be desirable to start prophylaxis if the patients were likely to suffer 2 further episodes (in addition to the present one) in the subsequent 5 years. He examined numerous criteria to see which identified those patients at risk. His conclusions were surprisingly simple. He found that if the patient had had one episode or more in the previous 5 years in addition to the present one, then he was likely to have 2 further episodes in the following 5 years. He also reported that at least 40% of unipolar patients would require prophylactic treatment. Of course, more than one episode in the previous 5 years would be a strong indication for starting prophylactic therapy. He could find no good criteria for discontinuing lithium therapy which should be continued indefinitely provided that patient’s adherence is satisfactory and serious adverse drug effects do not occur.

## Lithium and suicide prevention in unipolar depression

Whilst it is well established that recurrence of unipolar depression can be significantly reduced by adequate maintenance treatment, be it by antidepressants or lithium, it is strange, therefore, that the reduction in suicide rates over the years has been relatively modest (Coppen 2000a, b). A probable explanation of this modest reduction is that only a proportion of patients with unipolar depression are diagnosed, and only a proportion of those patients are treated, mostly with antidepressants. However, in contrast to a common belief, antidepressants do not reduce the risk of suicide (see below). If patients are adequately treated, however, preferably with lithium, one can observe a significant reduction of up to 75% in the suicide rate, as demonstrated by 18-year mortality and suicide rate in a group of 103 patients who attended a mood disorder clinic (Coppen and Farmer 1998). The majority of patients had unipolar depression which was severe and recurrent illness with a few dropouts during the first year of treatment. The standard treatment was sustained—release lithium given once-daily. Until 1982, the plasma lithium concentration measured approximately 12 h after dosage was maintained between 0.8 and 1.2 mmol/l. In 1982, the regiment was changed to maintain patients at the plasma lithium concentration between 0.6 and 0.8 mmol/l. Additional treatments, including antidepressants and neuroleptics, were administered as required.

In two other long-term follow-up studies in patients with affective disorders, patients had been treated for at least 1 year before recruitment and lithium levels were carefully monitored. In the first study, the International Group for the Study of Lithium treated Patients (IGSLI; ref. www.igsli.org) followed up 827 patients from 4 countries who attended lithium clinics for an average 7 years equalling 5600 patient years (Müller-Oerlinghausen et al. 1992). The overall suicide rate was 1.3 per 1000 patient years; the Standardised Mortality Rate (all causes) was 0.89. In other words, in contrast to an expected 2–3 fold increased all-cause mortality, the mortality of this long-term lithium-treated patient group did not differ from that of a matched cohort from the general population. This mortality reducing effect was observed in the unipolar patients as well as in those with bipolar or schizoaffective disorder. The other study involved mood disorder patients admitted to the main psychiatric hospital in Gothenburg, Sweden, between 1970 and 1991 who had received lithium for at least 1 year (Nilsson 1995). The suicide rate was 1.5 suicides per 1000 patient years for patients maintained on lithium treatment. For patients who had discontinued lithium, the suicide rate was 7.1 per 1000 patient years. Combining the three studies (11,085 patient years) yielded an unexpectedly low average suicide rate of 1.3 per 1000 patient years. (Bauer et al. 2015; Lewitzka et al. 2015a).

These rates contrast with the reports of suicide rates between 5.4 and 10.2 per 1000 patient years in long-term studies of patients not given maintenance treatment (Coppen and Farmer 1998). Furthermore, the IGSLI studies could also prove a reduction of the otherwise increased cardiovascular excess mortality of patients with affective disorders (Ahrens et al. 1995).

A systematic review of randomized, controlled trials of lithium reported that long-term lithium reduced the risk of suicide in mood disorders by about 75% as compared with placebo or other drugs (Cipriani et al. 2005).

An often heard argument by psychiatrists is that allegedly the antisuicidal effect of lithium refers exclusively to bipolar patients. However, an updated systematic review and meta-analysis on lithium in the prevention of suicide in mood disorders confirmed that also in unipolar depression lithium was associated with a reduced risk of suicide and the number of total deaths compared with placebo (Cipriani et al. 2013). Generally lithium compared with each active individual treatment showed superior efficacy in reducing the risk of deliberate self harm. Lithium tended to be generally better than the other active comparators, with small statistical variation between the results. The review concluded that lithium is an effective treatment for reducing the risk of suicide in people with mood disorders. Corresponding findings from very carefully documented unipolar patients of a specialized lithium clinic were reported by Guzzetta et al. (2007).

Lithium may exert its antisuicidal effects by reducing relapse of mood disorder, but additional mechanisms should also be considered because there is some evidence that lithium decreases aggression and possibly impulsivity, which might be another mechanism mediating the antisuicidal effect (Ahrens and Müller-Oerlinghausen 2001).

Maintenance treatment with antidepressants has been shown to be effective in terms of episode prevention when given for periods up to 5 years (Kupfer et al. 1992). There is a limitation with antidepressants; in that at least one-third of patients show a poor response to them. Moreover, it has been shown in controlled studies that antidepressants do not reduce the suicide risk in patients with mood disorders and that in some instances particularly the SSRIs can trigger de novo suicidal ideas and behaviour even in patients who never before experienced suicidal ideas (Fergusson et al. 2005; Bschor 2014; Stübner et al. 2010; Braun et al. 2016).

Maintenance treatment for depression may be continued for longer than 2 years if symptoms recur after discontinuation and in patients with late onset depression and delusional depression which is often highly recurrent with high risk of mortality by suicide and other causes. We refer to World Federation of Societies of Biological Psychiatry (WFSBP) Guidelines on Maintenance Treatment of Major Depressive that recommended “maintenance therapy is most commonly appropriate for recurrent patients, particularly when an episode prior to the present one has occurred in the last 5 years or when remission has been difficult to achieve. Maintenance treatment for 5–10 years or even indefinitely is recommended for those patients at greater risk, particularly when two or three attempts to withdraw medication have been followed by another episode within a year” (Bauer et al. 2015).

Lithium maintenance therapy is strongly recommended for the long-term management of selected patients with unipolar depression since there is strong evidence for its episode-preventing efficacy and unique anti-suicidal effects. The WFSBP Guidelines also do recommend the use of lithium as an effective maintenance treatment of recurrent depression.

## Conclusions

Lithium is an effective prophylactic treatment in selected patients with unipolar depression in its classic form. Most importantly, lithium is unique in being the only medicine with proven antisuicidal effects and hence a lethal disease is no longer lethal.

In our view, lithium prophylaxis is indicated in severe, clearly episodic unipolar depression that failed to respond to antidepressant medication.

We propose that in unipolar depression, the occurrence of 2 episodes of depression within 5 years is a practical criterion for starting lithium prophylaxis particularly in severe depression with psychotic features high suicidal risk and particularly in such patients in whom a bipolar background can be suspected. It seems self-evident that not only the subtype of depressive illness but also the psychosocial impact of the illness on the patient and the relatives must be adequately considered in making a sound shared decision.

In some cases, lithium prophylaxis may be recommended after a single episode of depression that is severe with high suicidal risk and continued life-long with regular and careful monitoring taking its specific profile of adverse drug reactions under proper consideration (Bauer and Gitlin 2016)

Lithium treatment has the advantage that it can be easily controlled to ensure adequate dosage and compliance and that other treatments such as antipsychotics or antidepressants can be added for some time if needed e.g., during break-through episodes. There is little doubt that recurrent mood disorders are most easily and satisfactorily treated in specialized mood disorder clinics. It is especially important that the initial assessment of a patient for long-term treatment be made by a specialist.

There is a need for large international RCTs comparing the efficacy of lithium prophylaxis with other recommended maintenance treatments of unipolar depression. Furthermore, it would be of eminent interest whether lithium would also possess acute anti-suicidal effects in patients with varying psychiatric diagnoses particularly those with unipolar depression (Lewitzka et al. 2015a, b).

In our view, the WHO should hasten to integrate the robust evidence on lithium’s antisuicidal effect demonstrated in various forms of affective disorder in its mental health action plan for the worldwide prevention of suicide.

In addition, there is strong evidence that lithium augmentation is effective in the management of patients with unipolar depression not responding to antidepressant medication. We, therefore, propose that lithium augmentation should be the first line treatment option among antidepressant augmentation strategies, particularly after two different antidepressants have been tried unsuccessfully (Tundo et al. 2015)

Finally, lithium is also an effective continuation treatment following ECT.
